# Synthesis of silver nanoparticles using a *Mentha spicata* extract and evaluation of its anticancer and cytotoxic activity

**DOI:** 10.7717/peerj.8142

**Published:** 2019-12-09

**Authors:** Yuridia Torres-Martínez, Eder Arredondo-Espinoza, Carlos Puente, Omar González-Santiago, Nayely Pineda-Aguilar, Isaías Balderas-Rentería, Israel López, Mónica A. Ramírez-Cabrera

**Affiliations:** 1 Universidad Autónoma de Nuevo León, UANL, Facultad de Ciencias Químicas, Laboratorio de Farmacología Molecular y Modelos Biológicos, Monterrey, Nuevo León, Mexico; 2 Universidad Autónoma de Nuevo León, UANL, Facultad de Ciencias Químicas, Laboratorio de Materiales I, San Nicolás de los Garza, Nuevo León, Mexico; 3 Universidad Autónoma de Nuevo León, UANL, Centro de Investigación en Biotecnología y Nanotecnología (CIBYN), Laboratorio de Nanociencias y Nanotecnología, Apodaca, Nuevo León, Mexico; 4 Centro de Investigación en Materiales Avanzados, S.C. (CIMAV), Unidad Monterrey, Apodaca, Nuevo León, Mexico

**Keywords:** Silver nanoparticles, Anticancer, Citotoxicity, *Mentha spicata*

## Abstract

In this study, silver nanoparticles (NP) were synthesized by two methods: using an aqueous extract of *Mentha spicata* leaves and using citrate ions as stabilizing agent, and the cytotoxicity and anticancer activity of both NP were evaluated in vitro. The particles synthesized with the aqueous extract were spherical with a size ranging from 15 to 45 nm. These NP decreased cell viability in all of the cells studied; however, the IC_50_ could only be estimated in the Chang liver cells (IC_50_ = 21.37 µg/mL). These particles also decreased the generation of reactive oxygen species in Chang and SiHa cells. Additionally, the dispersions decreased the activity of caspase-3. There was no significant difference between the biological activities of the NP obtained with the aqueous extract and the NP synthesized using citrate ions. This study showed that an aqueous extract of *M. spicata* is an excellent alternative for the synthesis of silver NP. These NP showed cytotoxicity and anticancer activity in vitro. Although more experiments are required, the cell death occurs probably through a mechanism different from apoptosis.

## Introduction

The incidence and mortality of cancer are growing rapidly worldwide for many reasons. Aging and population growth are among the main causes; however, environmental factors, such as tobacco smoking, urbanization and its associated pollution, and changing diet patterns have also been considered responsible for this phenomenon (Vineis & Wild, 2014; You & Henneberg, 2018). According to GLOBOCAN, 18.1 million new cases and 9.6 million deaths were estimated around the world during 2018 (Bray et al., 2018). Considering the high mortality rate of cancer, and due to the severe adverse effects of most anticancer drugs, a search for new anticancer molecules is urgent.

Plants represent an important source for the development of new drugs, due to the several important biological activities found when tested in vitro. In the case of *Mentha spicata*, activities such as fungicidal, antibacterial, anticancer, antiemetic, and analgesic have been reported (Govindarajan et al., 2012; Tayarani-Najaran et al., 2013; Sharma et al., 2014; Houicher et al., 2016; Mahboubi, 2017). Thanks to these activities, the extracts of this plant merit more research.

On the other hand, several plant extracts have been used in recent years to produce metallic nanoparticles (NP) instead of producing these materials through chemical procedures. This approach is known as green synthesis and some of its advantages are a lower production cost, a reduction in the use of toxic chemicals, a reduction in the reaction steps needed during synthesis, and lower energy consumption (Mittal, Chisti & Banerjee, 2013). An additional advantage is that the biological activities of the metallic NP can be improved due to the biomolecules in the nanoparticle surface; some examples are the antimicrobial and anticancer properties.

Among metallic NPs, those of silver (AgNP) and gold (AuNP) have shown important in vitro biological activities, such as anticancer and antimicrobial effects (Kim & Hyeon, 2014). Green synthesis of this kind of materials using biologically active plant extracts could improve their biological activities or even reduce their toxicity. The objective of this study was to synthesize AgNP using *M. spicata* extracts (AgNP-MS) and citrate ions (AgNP-C) as stabilizing agents and to evaluate the in vitro cytotoxic and anticancer activities of the two nanoparticle dispersions.

## Methods

### Synthesis and characterization of AgNP

#### Aqueous extract preparation

A total of 5 g of clean, fresh cut leaves of *M. spicata*, which were commercially acquired in a local supermarket, and 50 mL of water were placed in a flask (Pyrex), boiled for 5 min and then filtered with a Whatman filter paper. The final volume of the extract was completed to 50 mL with deionized water and was subsequently stored at 4 °C until use.

#### Quantification of total polyphenolic content

This analysis was carried out with the Folin–Ciocalteu colorimetric method, which was described by Singleton, Orthofer & Lamuela-Raventós (1999). Briefly, 2.5 mL of Folin–Ciocalteu reagent was added to 500 µL of the aqueous extract of *M. spicata*; after 5 min, two mL of Na_2_CO_3_ (7.5%) was added. Finally, after 2 h in the absence of light, the absorbance of this mixture was determined at 760 nm using gallic acid as the standard.

#### Synthesis of silver nanoparticles

In a regular synthesis of *M. spicata-*based nanoparticles (AgNP-MS), two mL of AgNO_3_, two mL of the plant extract, and 46 mL of water were placed in a 250 mL Erlenmeyer flask (Pyrex, New York, NY, USA). This mixture was placed in a conventional microwave oven for 1 min at 1,275 W. The color changed from light yellow to orange, which is indicative of the formation of AgNP.

The synthesis of silver nanoparticles capped with citrate ions (AgNP-C) has been thoroughly studied by different authors, including our research group. In a regular synthesis, 8 mL of 73 mM AgNO_3_ and 30 mL of a 5 mM sodium citrate solution are mixed with 61 mL of deionized water, and then 1 mL of an 8.0 mM solution of NaBH_4_ is added dropwise under vigorous stirring (Lopez et al., 2015).

#### Characterization of nanoparticles

To analyze the optical properties of the NP, a UV-1800 UV spectrophotometer (Shimadzu, Kyoto, Japan) was used. To characterize the morphology of the dispersions, a dynamic light scattering (DLS) analyzer (Zetatrac NPA152 Microtrac) and a field emission scanning electron microscope (FE-SEM) (FEI Nova NanoSEM 200) were also used.

### Cytotoxic and anticancer activities

#### Cell culture

To evaluate cytotoxic activity, Chang liver cells (CCL-13 ATCC) were used, while for the evaluation of anticancer activity, SiHa (HTB-22D ATCC) and colon cancer (HTB-29 ATCC) cells were used. The cell cultures were grown in Eagle’s minimum essential medium (EMEM; Sigma Aldrich, St. Louis, MO, USA) (four mL) supplemented with 10% fetal bovine serum (FBS; GIBCO, Waltham, MA, USA) and 1% penicillin/streptomycin (Sigma Aldrich, St. Louis, MO, USA). The growth conditions were 37 °C at 5% CO_2_.

#### Cell viability assay

Viability of the cells was determined using a colorimetric assay that uses the cell proliferation reagent, WST-1 (Roche, Mannheim, Germany). The procedure was performed according to the recommendation of the manufacturer (Roche, Mannheim, Germany). Cells (1 × 10^4^) were placed in 96-well plates (Corning, Corning, NY, USA) until they reached confluence, then they were exposed to AgNP-MS or AgNP-C. The concentrations of the NP were 3.12, 6.25, 12.5, 25, 50, 100, and 200 µg/mL. The final volume was of 100 µL for each well, and the time of exposure was 24 h at 37 °C with 5% CO_2_. After incubation, the plates were washed twice with 100 µL of PBS, then 100 µL of EMEM with WST-1 at 5% was added. Plates were incubated for 2 h in the previously mentioned conditions and optical density was determined using a plate reader (ELx800) at 450 nm. Vincristine (Sigma Aldrich, St. Louis, MO, USA) was used as a positive control for anticancer activity; Triton X-100 at 1% was used as a positive control for cytotoxic activity, and culture medium was the negative control.

### Intracellular production of reactive oxygen species

The oxidation-sensitive dye, 2′-7′-Dichlorodihydrofluorescein diacetate (DCF-DA; Sigma Aldrich, St. Louis, MO, USA), was used to determine the production of reactive oxygen species (ROS). Chang and SiHa Cells (1 × 10^4^) were placed in 96-well plates and incubated for 24 h in the previously mentioned conditions. Then AgNP-MS and AgNP-C were added. The final concentrations of the nanoparticle dispersions were 3.12, 6.25, 12.5, 25, 50, 100, and 200 µg/mL. Thereafter, 100 µL of DCF-DA was added to each well. Xanthine-oxidase (Sigma Aldrich, St. Louis, MO, USA) was used as a positive control (final concentration, 31.25 µL), and cells in culture were used as a negative control. The final volume was of 200 µL for each assay. The fluorescence, which is proportional to the ROS produced, was measured in a fluorometer (Fluoroskan Ascent) at an excitation wavelength of 485 nm and an emission wavelength of 530 nm. The fluorescence measurement was to 24 h.

### Apoptotic activity

The apoptotic activity of the NP was evaluated by measuring the activity of caspase-3, which is an Asp-Glu-Val-Asp (DEVD) specific protease. A commercial kit was used, and the procedure was done according to the manufacturer’s instructions. Briefly, after cells were exposed to AgNP-MS and AgNP-C for 24 h in the previously mentioned conditions, they were lysed by the addition of 50 µL of 1× lysis buffer for 30 min at 4 °C, then they were centrifuged at 5,000×*g* for 5 min. The supernatant was collected and the protein content was quantified with the Bradford method. Later, 0.5 µg of protein were incubated with Z-DEVD–R110 substrate for 30 min at room temperature.

### Statistical analysis

The IC_50_ values were estimated with a log regression and expressed as the mean ± standard deviation of three independent experiments. The difference among groups was determined with Student’s *t*-test and ANOVA as corresponded. The statistical software NCSS12 was used for all analyses. A *p* < 0.05 was considered statistically significant.

## Results

### Synthesis and characterization of AgNP-MS and AgNP-C

**Figure 1:**
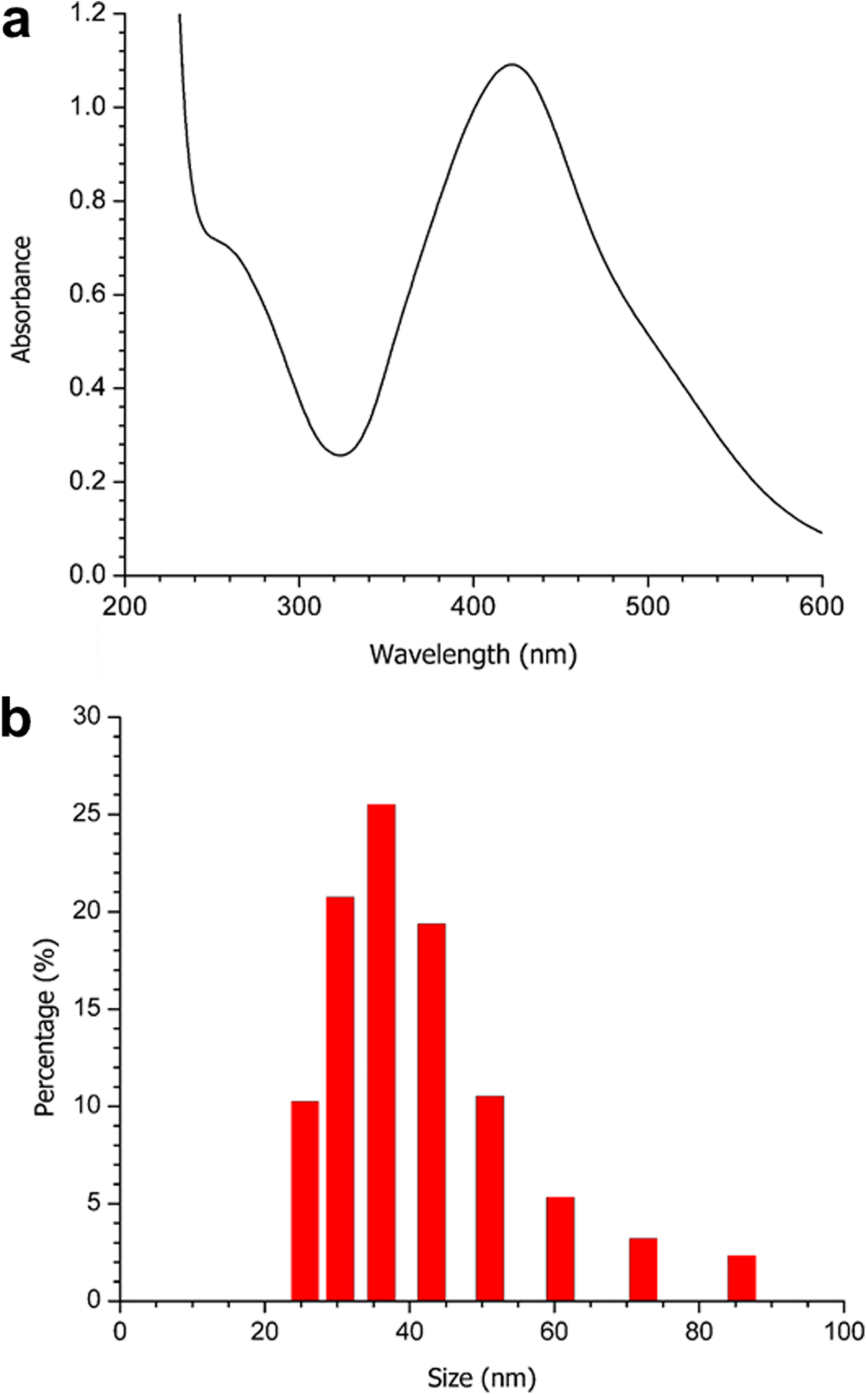
(A) UV-Vis spectrum, and (B) DLS analysis of the obtained AgNP-MS.

Strong absorption of electromagnetic waves is exhibited by metal NP, caused by the coherent oscillation of their electrons. This is known as surface plasmon resonance (SPR). The absorption band of the AgNP-MS SPR was observed at 422 nm (Fig. 1A), which is characteristic for silver nanospheres.

**Figure 2:**
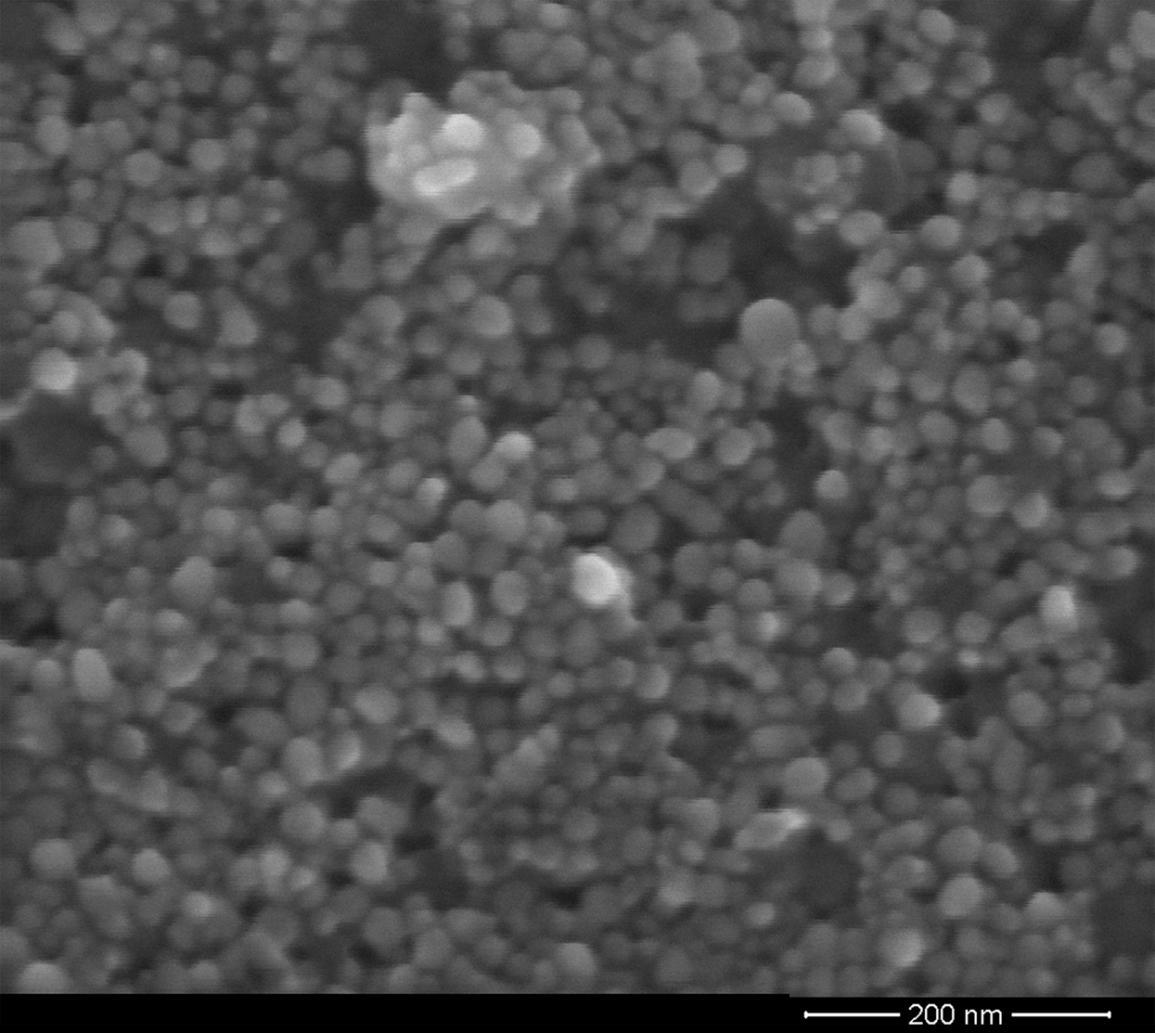
FE-SEM micrograph of the AgNP-MS dispersion.

According to the DLS analysis, the NP synthesized showed a size between 15 and 37 nm, with 20 nm being the size with the highest percentage of appearance (Fig. 1B). On the other hand, the FE-SEM images of the synthesized AgNP-MS were mainly spherical in shape (Fig. 2) with a size range between 30 and 45 nm, with 36 nm as the average size.

### Quantification of total polyphenolic content

The aqueous extract of *M. spicata*, which was used for the synthesis of AgNP-MS, had a polyphenol content of 5.95 mg/g of leaves, in gallic acid equivalent, according to the Folin–Ciocalteu analysis.

### Cytotoxic evaluation of AgNP-MS and AgNP-C

**Figure 3:**
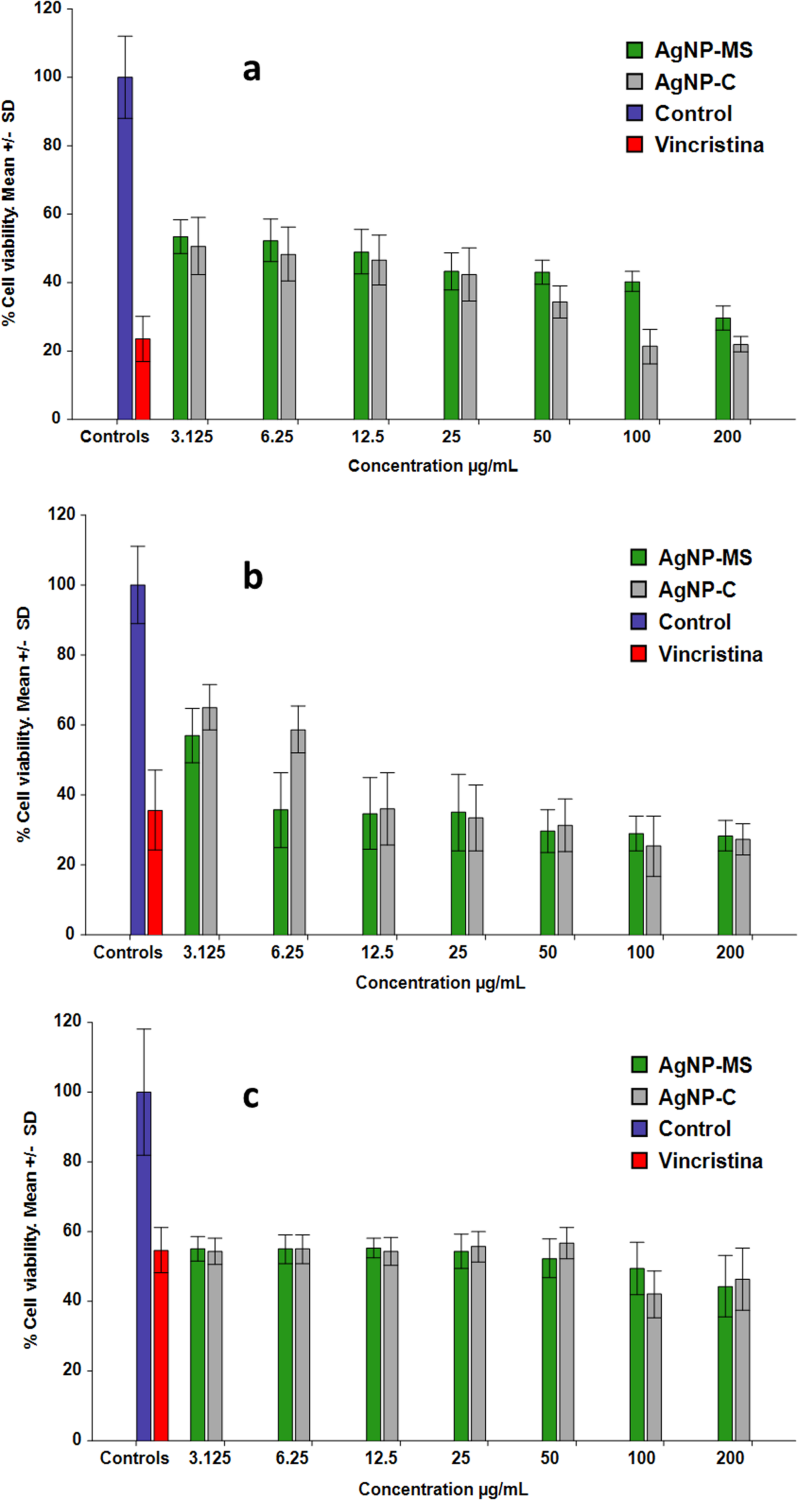
Cell viability assay. (A) Chang liver cells, (B) SiHa cells, and (C) Colon cancer cell.

The AgNP-MS inhibited the proliferation of Chang liver cells in the concentrations studied. The percentage of cell viability ranged from 53.3% to 30.7% (*p* = 0.052) for concentrations in the range of 3.12 to 200 µg/mL (Fig. 3A), and the estimated IC_50_ was 21.37 ± 10.96 µg/mL. Similarly, AgNP-C showed inhibition of cell proliferation with the percentage of cell viability ranging from 51.7% to 22.0% (*p* = 0.05) for the same range of concentrations (Fig. 3A). The estimated IC_50_ was 13.25 ± 7.14 µg/mL for the latter dispersion. There was no significant difference between the IC_50_ values of AgNP-MS and AgNP-C (*p* = 0.18).

### Anticancer activity of AgNP-MS and AgNP-C

The AgNP-MS inhibited cell proliferation of both SiHa and HT-29 cells. In the case of SiHa cells, the percentage of cell viability was from 57.0% to 28.2% (*p* = 0.26) for concentrations ranging from 3.12 to 200 µg/mL (Fig. 3B). For HT-29 cells, the percentage of cell viability was 55.0% to 44.3% (*p* = 0.78) (Fig. 3C) for the same range of concentrations. All studied concentrations were significantly different with respect to control; however, as there was no significant difference among the concentrations, the IC_50_ in SiHa and HT-29 cells were not estimated.

The AgNP-C also showed inhibition of cell proliferation in both cell types. In the case of SiHa cells, the percentage of cell viability was 65.0% to 27.3% (*p* = 0.015) (Fig. 3B) with an IC_50_ of 16.49 ± 9.96 µg/mL. For HT-29 cells, the percentage of cell viability was 54.3% to 46.3% (*p* = 0.64) for the same range of concentrations (Fig. 3C). Similarly to AgNP-MS, the IC_50_ were not estimated due to the lack of significant difference among the concentrations in both cell lines.

### ROS production

**Figure 4:**
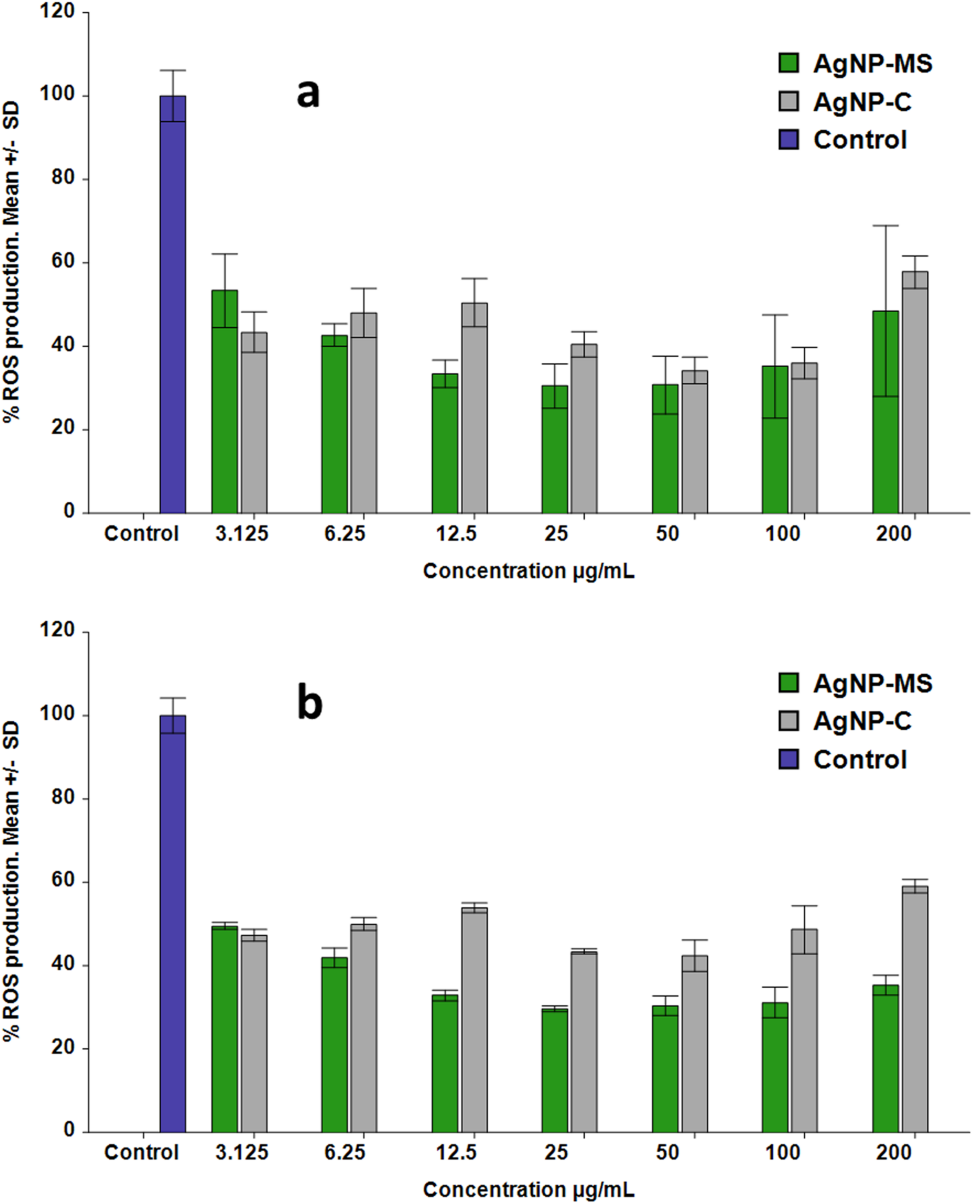
Production of ROS in cells exposed to AgNP-MS and AgNP-C. (A) Chang liver cells, and (B) SiHa cells.

Reactive oxygen species production was evaluated in Chang liver and SiHa cells, and it was expressed as a percentage respect to cells without treatment (100%). In the case of the former cell type, AgNP-MS produced lower levels of ROS than the control with values from 30.5% to 53.3% for the concentrations ranging from 3.12 to 200 µg/mL. There was no significant difference among the concentrations studied (*p* > 0.05); however, all concentrations were different with respect to control (*p* < 0.01). On the other hand, AgNP-C produced ROS ranging from 34.2% to 57.8% (*p* ≥ 0.05) in the same concentration range, there was also no significant difference among the studied concentrations; however, all were different with respect to control (*p* ≤ 0.01) (Fig. 4A).

In the case of the SiHa cells, AgNP-MS produced lower levels of ROS than the control, these were from 29.7% to 49.5% for the concentrations ranging from 3.12 to 200 µg/mL, there was no significant difference among the concentrations; however, all were different respect to control (*p* < 0.01). AgNP-C also produced lower levels of ROS than control, the values range from 42.4% to 59.0% for the same concentration range (Fig. 4B). Once again, there was no significant difference among concentrations (*p* > 0.05) but it does with respect to the control (*p* < 0.01).

### Apoptotic activity

**Figure 5:**
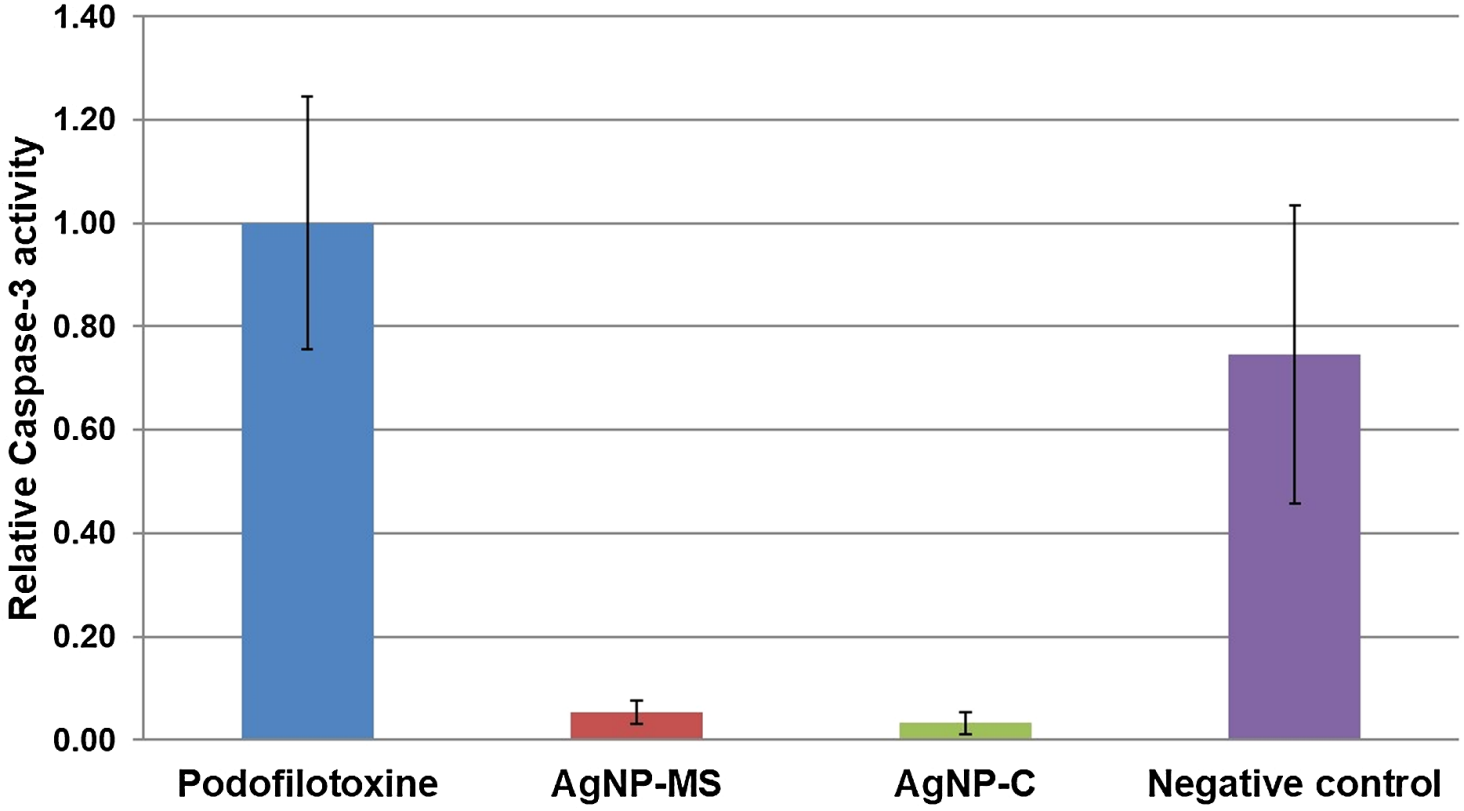
Activity of caspase-3 in SiHa cells exposed to AgNP-MS and AgNP-C.

Caspase-3 activity was measured in SiHa cells after 24 h of exposure to AgNP-MS or AgNP-C. Our results indicate that both NP do not induce the apoptosis process, the activity of caspase-3 was minimal compared to the negative control and podophyllotoxin; this suggests that the possible mechanism of cell death is through another mechanism such as necrosis (*p* < 0.01) (Fig. 5).

## Discussion

In this study, silver NP were synthesized using an aqueous extract of *M. spicata* in order to evaluate their cytotoxic and anticancer activities. The obtained results showed that the aqueous extract of this plant is an efficient and greener alternative for the synthesis of spherical silver NP. In previous studies, the use of different plant extracts has proved to be a good alternative for the synthesis of silver or gold NP due to the reducing and stabilizing capabilities of the secondary metabolites that can be extracted by boiling in water any kind of plant tissue, from roots to flowers. To date, there are more than 50 plant species and cultivars that have been used for the synthesis of metallic NP, mainly of silver and gold (Mittal, Chisti & Banerjee, 2013; Hanan et al., 2018).

The AgNP-MS showed important cytotoxic activity (IC_50_ = 21.37 µg/mL) in Chang liver cells. This is the first study that evaluates this activity using this type of cells. Others studies, using the same nanoparticle morphology (with diameters between 30 and 150 nm), had reported important antimicrobial activity and low antifungal activity (Ahmad et al., 2016). Our results did not show a significant difference between AgNP-MS and AgNP-C in their cytotoxic activity, even though the latter has a smaller size (10 nm) and a different stabilizing agent (citrate ions), which is the part of the nanoparticle that interacts directly with its environment, in this case, with cells (Mailänder & Landfester, 2009; Ferreyra Maillard et al., 2018). Silver NP synthesized with different plant extracts have proven different cytotoxic profiles with an IC_50_ ranging from 10 to 1,000 µg/mL. The most commonly used cell strains for this analysis are Lymphocyte, HBL100, normal colon, HaCaT, HSFs, W1-38, MDCK, VERO, CV-1, and 3T3-L1 cell lines (Hanan et al., 2018).

In the case of anticancer activity, both AgNPs decreased cell viability in the studied cancer cell lines. According to the literature, anticancer activity is highly variable among NP that have been synthesized with different plant extracts; the reported IC_50_ ranges from 1 to 2,010 µg/mL. The most studied cells are A549, HT-29, MCF7, HeLa, among others. On the other hand, there is no significant difference in the cytotoxic activity between AgNP-MS and AgNP-C. Although this lack of difference is not clear, it could be attributed to a high variability in the three AgNP-MS assays. This variability could be due to the inhomogeneous capping of the NP, as the aqueous extract of *M. spicata* could have several chemical compounds in addition to polyphenols, which can react and bind to the external layer of the NP. This means that particles with different chemical compounds in its external layer could be present and so influence cytotoxicity and anticancer activity. Further studies are required to confirm this hypothesis.

The AgNP-MS produced a decrease in the generation of ROS. This activity could be due to the content of polyphenolic compounds, which was quantified in the aqueous extract of *M. spicata*. These compounds have been detected on the surface of silver NP obtained using plant extracts (Lopez et al., 2015). Previous studies have also reported that this plant contains polyphenols and flavonoids with a considerable antioxidant activity (Abootalebian et al., 2016; Abdul Qadir et al., 2017). In the same way, AgNP-C also produced a decrease in the generation of ROS, which is in contradiction with the literature (Nguyen et al., 2013; Bastos et al., 2016). The discrepancies between different studies could be due to different methods or cells used to evaluate the ROS produced by AgNP.

We evaluated caspase-3 activity in order to determine the possible mechanism of cell death. Our results indicate that both NP do not induce the apoptosis process, the activity of caspase-3 was minimal compared to the negative control and podophyllotoxin, this suggests that the possible mechanism of cell death is through another mechanism such as necrosis. Nevertheless, the latter must be further analyzed and compared with others assays such as flow cytometry using 7-AAD dye (Kumar et al., 2015) or lactate dehydrogenase assay (Chan, Moriwaki & De Rosa, 2013). The lower production of ROS supports this possible mechanism as it is well known that higher production of ROS induce apoptosis (Merad-Boudia et al., 1998; Dhanasekaran & Reddy, 2008; Circu & Aw, 2010). Another possible mechanism of cell death could be through induction of autophagy (Wang et al., 2017; Li & Ju, 2018); however, more studies are needed to prove this possibility.

## Conclusions

Spherical silver NP were successfully synthesized with the aqueous extract of *M. spicata*. This method of synthesis is a greener alternative for the synthesis of this kind of material. These NP have similar in vitro cytotoxic and anticancer activities to those of silver NP capped with citrate ions. The cell death induced by synthesized NP may be through a mechanism different from apoptosis.

## Supplemental Information

10.7717/peerj.8142/supp-1 Supplemental Information 1 Raw data HT-29 cells.

10.7717/peerj.8142/supp-2 Supplemental Information 2 Raw data SiHa Cell.

## Additional Information and Declarations

### Data Availability

The following information was supplied regarding data availability: Data are available as Supplemental Files.
